# Mediodorsal Thalamic Neurons Mirror the Activity of Medial Prefrontal Neurons Responding to Movement and Reinforcement during a Dynamic DNMTP Task

**DOI:** 10.1523/ENEURO.0196-17.2017

**Published:** 2017-10-13

**Authors:** Rikki L.A. Miller, Miranda J. Francoeur, Brett M. Gibson, Robert G. Mair

**Affiliations:** Department of Psychology, University of New Hampshire, Durham, NH 03824-2602

**Keywords:** Adaptive behavior, decision-making, higher order thalamic nuclei, rat, spatial coding, tetrode

## Abstract

The mediodorsal nucleus (MD) interacts with medial prefrontal cortex (mPFC) to support learning and adaptive decision-making. MD receives driver (layer 5) and modulatory (layer 6) projections from PFC and is the main source of driver thalamic projections to middle cortical layers of PFC. Little is known about the activity of MD neurons and their influence on PFC during decision-making. We recorded MD neurons in rats performing a dynamic delayed nonmatching to position (dDNMTP) task and compared results to a previous study of mPFC with the same task (Onos et al., 2016). Criterion event-related responses were observed for 22% (254/1179) of neurons recorded in MD, 237 (93%) of which exhibited activity consistent with mPFC response types. More MD than mPFC neurons exhibited responses related to movement (45% vs. 29%) and reinforcement (51% vs. 27%). MD had few responses related to lever presses, and none related to preparation or memory delay, which constituted 43% of event-related activity in mPFC. Comparison of averaged normalized population activity and population response times confirmed the broad similarity of common response types in MD and mPFC and revealed differences in the onset and offset of some response types. Our results show that MD represents information about actions and outcomes essential for decision-making during dDNMTP, consistent with evidence from lesion studies that MD supports reward-based learning and action-selection. These findings support the hypothesis that MD reinforces task-relevant neural activity in PFC that gives rise to adaptive behavior.

## Significance Statement

MD is a higher-order thalamic nucleus that plays an important role in associative learning and adaptive decision-making. MD has prominent reciprocal connections with PFC and receives other inputs from subcortical systems that signal information about reward, emotions, sensorimotor function, and behavioral state. We report that MD neurons exhibit temporal patterns of activity similar to mPFC in rats performing a dDNMTP task. MD primarily signaled information about movement and reinforcement important for decision-making in dDNMTP but lacked other types of responses prominent in PFC during this task. We hypothesize that MD is organized to reinforce PFC neuronal responses that signal task-relevant information essential for adaptive behavior.

## Introduction

The mediodorsal thalamic nucleus (MD) interacts with prefrontal cortex (PFC) to support adaptive goal-directed behavior (Parnaudeau et al., 2013; Browning et al., 2015; Mitchell, 2015; Bolkan et al., 2017). MD lesions disrupt flexible association of actions with outcomes in decision-making, including delayed conditional discrimination, object-in-place, and object-reward tasks (Mair et al., 2015; Mitchell, 2015), and affect the ability to update reward value with task manipulations and in reversal and multiple-option learning (Mitchell et al., 2007; Ostlund and Balleine, 2008; Izquierdo and Murray, 2010; Bradfield et al., 2013; Chakraborty et al., 2016).

MD receives driver (layer 5) and more extensive modulatory (layer 6) inputs from PFC and provides dense focal projections to middle layers and sparser diffuse projections to layer I of PFC (Goldman-Rakic and Porrino, 1985; Giguere and Goldman-Rakic, 1988; Groenewegen, 1988; Kuroda et al., 1996, 1998; Xiao et al., 2009). MD also receives inputs from amygdala, reward- and motor-processing areas of pallidum, and visceral and arousal systems in midbrain and brainstem (Kuroda and Price 1991a,b; O’Donnell et al., 1997; McFarland and Haber, 2002; Krout et al., 2002; Tripathi et al., 2013; Root et al., 2015; Timbie and Barbas 2015; Vertes et al., 2015). In the rat, MD consists essentially of thalamocortical neurons, with few (≤1%) interneurons (Kuroda and Price, 1991a; Kuroda et al., 1992, 1998). Thus rat MD appears to be organized as a transthalamic gate (Sherman and Guillery, 2011), where thalamocortical neurons driving PFC activity integrate driver and modulatory inputs from PFC with signals from systems mediating reward and reward-guided responding.

There is surprisingly little known about what information is represented in MD and how it influences prefrontal function. Early studies emphasized activity sustained throughout memory delays in MD and PFC related to sensory cues and motor responses hypothesized to represent working memory in monkeys performing spatial memory tasks (Fuster and Alexander, 1971; Watanabe and Funahashi, 2004a,b, 2012). Bolkan et al. (2017) reported that optogenetic inhibition of MD to mPFC projections in mice impairs T-maze delayed nonmatching to position (DNMTP) performance when applied during 60-s (but not 10-s) memory delays. Nevertheless, questions remain about whether delay-period activity represents information held in working memory for neurons in rodent mPFC (Cowen and McNaughton, 2007; Horst and Laubach, 2012) or MD (Han et al., 2013).

Recordings of mPFC neurons in awake, behaving rats have revealed a wide range of task-related responses including activity related to preparation, sensorimotor cues, movements and other actions, anticipated and actual reinforcement, errors, behavioral strategy, and spatial context, as well as delay-related activity in memory tasks (Jung et al., 1998; Pratt and Mizumori, 2001; Hok et al., 2005; Euston and McNaughton, 2006; Cowen and McNaughton, 2007; Rich and Shapiro, 2009; Totah et al., 2009, 2013; Horst and Laubach, 2012; Powell and Redish, 2014, 2016). The limited available evidence suggests that MD neurons in the rat may represent a similarly broad range of task-related information. This includes reports that MD neurons represent information about behavioral choice, reinforcement location, and choice outcome during spatial delayed alternation (Han et al., 2013); olfactory and spatio-motor information during two-alternative odor discrimination (Courtiol and Wilson, 2016); and conditioned (but not spontaneous) motor responses and reward value of conditioned stimuli during associative learning (Oyoshi et al., 1996). The range of task-related information represented in mPFC and MD in the rat seems consistent with the broad effects of lesions in mPFC (Dalley et al., 2004; Chudasama, 2011) and MD (Bradfield et al., 2013; Mitchell, 2015) on goal-directed behavior.

To understand the messages relayed between MD and mPFC during adaptive decision-making, we recorded MD neurons in rats performing a dynamic DNMTP (dDNMTP) task, using procedures and apparatus previously found to produce neuronal responses in mPFC related to preparation, movement, lever press actions, reinforcement, errors, memory delays, and spatial location of behavioral events (Onos et al., 2016). Our goal is to understand what information is represented in MD during dDNMTP and how this compares with results for mPFC. We found a more limited set of event-related response types in MD than mPFC, with a preponderance of movement- and reinforcement-related activity and a lack of responses related to preparation, lever presses, and memory delay. MD neurons exhibited temporal patterns of activity that mirrored the firing patterns of homologous response types in mPFC, consistent with the reciprocal connections of these areas.

## Materials and Methods

### Animal subjects

Nine male Long-Evans rats were obtained at 3 wks of age from Harlan Laboratories and housed singly on a 12:12 h light:dark cycle with training and recording studies during the light phase. Rats were given *ad libitum* access to food and water until they reached a weight of 250 g, when a water restriction schedule began so that water could be used as a positive reinforcer. Rats received water during training sessions and for 30 min of free access near the end of light cycle or for 1 h of free access on days when they were not trained. This research was conducted in strict accordance with the Guide for the Care and Use of Laboratory Animals of the National Institutes of Health. The protocol was approved by the institutional animal care and use committee at the University of New Hampshire.

### Behavioral training

**Figure 1.**
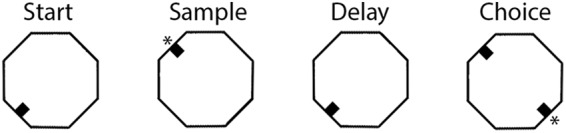
The dynamic DNMTP task consisted of a sequence of 4 lever presses trained in an octagonal chamber equipped with 4 retractable levers centered on walls 90° apart. Trials began with the base lever (randomly selected for each trial) extended for the start response. This retracted when pressed, and the sample lever extended (randomly selected 90° to the left or right of the base). Reinforcement (indicated by *) was delivered to the spout immediately above the sample lever when it was pressed. The sample lever was then retracted and the base lever extended. This was retracted on the first press after the memory delay, causing levers 90° to the left and right to extend. Reinforcement was delivered when rats then pressed the lever opposite the sample. There was a 5-s intertrial interval.

Rats were trained in clear polycarbonate octagonal arenas, 61 cm in diameter. Retractable levers (ENV-112CM, Med Associates) were centered on 4 walls 90° apart (N, E, S, W), each with a stimulus light (ENV-221M, Med Associates) and drinking spout above to signal and deliver water reinforcement by activation of a miniature solenoid valve (LFAA1201518H, The Lee Co.). The arena used for recording studies was located in a Faraday cage with a screen door that provided ambient illumination and many visible external cues. The behavioral apparatus was controlled by a PC interface (Med Associates) in an adjacent room.dDNMTP trials comprised a sequence of 4 lever press responses (start, sample, delay, choice) on 3 of the retractable levers in a “T” configuration (Fig. 1). Trials began with the base lever (the stem of the “T”) extending for the start response. This retracted when pressed and was followed by the sample lever extending, randomly selected 90° to the left or right of the base. This retracted when pressed. Reinforcement was delivered to the spout immediately above the sample lever, and the base lever was extended for the delay response. Reinforcement was signaled by panel lights located immediately above drinking spouts and by sounds of miniature solenoid valves (mounted on the outside wall of the chamber) that delivered water. The base lever retracted with the first press after the memory delay and 2 levers, 90° to the left and right of the base, extended for the choice response. Reinforcement was then delivered, and all levers retracted when the rat pressed the lever not extended for the sample press (i.e. nonmatching to sample position). When this was the first lever pressed, the response was scored as correct. When rats pressed the incorrect lever first, the trial was scored as an error and rats were allowed to continue until they pressed the nonmatching lever and received reinforcement. This correction procedure (allowing rats to proceed until they received reinforcement for pressing the nonmatching lever) was followed to maintain the nonmatching strategy and avoid positional response biases.

For most sessions, the base lever location was randomly selected for each trial from 2 levers on opposite sides of the arena, alternating paired locations (N/S vs. E/W) between successive sessions. Selecting base levers in this manner avoids confounding behavioral events with specific locations and is the main distinction between dDNMTP and traditional DNMTP tasks. At the start of the study, a few sessions were conducted where the base lever location was randomly sampled from all 4 levers. Reinforcement (following sample and correct choice responses) consisted of two 0.1-s (0.1-mL) pulses of water, 1.0 s apart. The memory delay was 3 s on all trials. There was a 5-s intertrial interval between delivery of choice reinforcement at the end of one trial and extension of the start (base) lever to begin the next trial.

### Electrophysiological recording

Electrophysiological activity was recorded from an array of 4 tetrodes, through a head stage and tether (Neuralynx) connected through a low-torque slip-ring commutator (Dragonfly Research and Development) to a Neuralynx Digital Lynx SX high-density electrophysiology recording system. Tetrodes consisted of four 17.8-μm platinum iridium microwires (California Fine Wire) twisted using a Tetrode Spinner v. 2 (Neuralynx) and contained in a stainless steel cannula (from which tetrodes extended into brain tissue). Five rats had a single cannula of 4 tetrodes in one hemisphere (3 left, 2 right), and four had a cannulae in each hemisphere each containing 2 tetrodes. The cannulae were soldered to a central pin in an 18-pin Mill-Max socket with a sliver ground wire and the 16 microwires of the tetrodes attached to the other pins. Tetrode arrays were fastened through a poly(methyl methacrylate) base to a tripod of two 56 × 15-mm stainless steel base screws that screwed into threaded sockets glued to the skull. This allowed us to lower tetrode arrays incrementally between recording sessions. Before implantation, each microwire electrode was tested and plated with platinum black to lower impedances to a target ≤200 kΩ at 1 kHz using a Nano-Z (Neuralynx). Recorded signals were amplified and processed using Cheetah data acquisition software. Digital signal processing low-cut and high-cut filters were set to 600–6000 Hz.

### Surgical procedures

Rats were anesthetized with an intramuscular injection of ketamine (85 mg/kg) and xylazine (8.5 mg/kg) and placed in a stereotaxic instrument, and tetrode arrays were implanted using aseptic techniques 6.2 mm anterior to the intra aural line (IA), 5.2 mm dorsal to IA. To compensate for differences from atlas rats, AP coordinates were measured from both IA and bregma and DV coordinates from both IA and the surface of cortex overlying thalamus. Where these measures differed (in all cases <0.5 mm), they were averaged to determine the stereotaxic site for implanting tetrode arrays. The distance lateral from midline was varied from 0.4 to 1.2 mm to sample neurons in different areas of MD. A small opening for the tetrode array was made in the skull, and holes were drilled for 0-80 stainless steel skull screws, to which the sockets securing the base screws of the tetrode array were attached with Grip cement (Dentsply). Butorphanol (0.2 mg/kg subcutaneously) was administered at the end of surgery for postsurgical analgesia.

### Histology

Tetrode tracks were marked by passing 100 µV current for 30 s using an A365 constant current stimulus isolator (WPI) at the end of experiments. Rats were killed 3 d later under deep anesthesia (100 mg/kg ketamine, 10 mg/kg xylazine intramuscular) by transcardiac perfusion of physiologic saline followed by 4% (vol/vol) neutral buffered formalin. Tetrodes were backed out and removed to minimize postmortem damage, and brains were removed and immersed in 30% sucrose, 4% neutral buffered formalin until they were ready to be sectioned. Tissue was blocked in the flat skull position using an RBM 4000C brain matrix (ASI Instruments), sectioned frozen in the coronal plane at 50 µm, and stained with thionin. Tissue was examined to identify the course of the tetrode track. The locations of individual neurons recorded were inferred based on the number of turns the arrays had been advanced when they were recorded.

### Experimental design and data analyses

**Figure 2.**
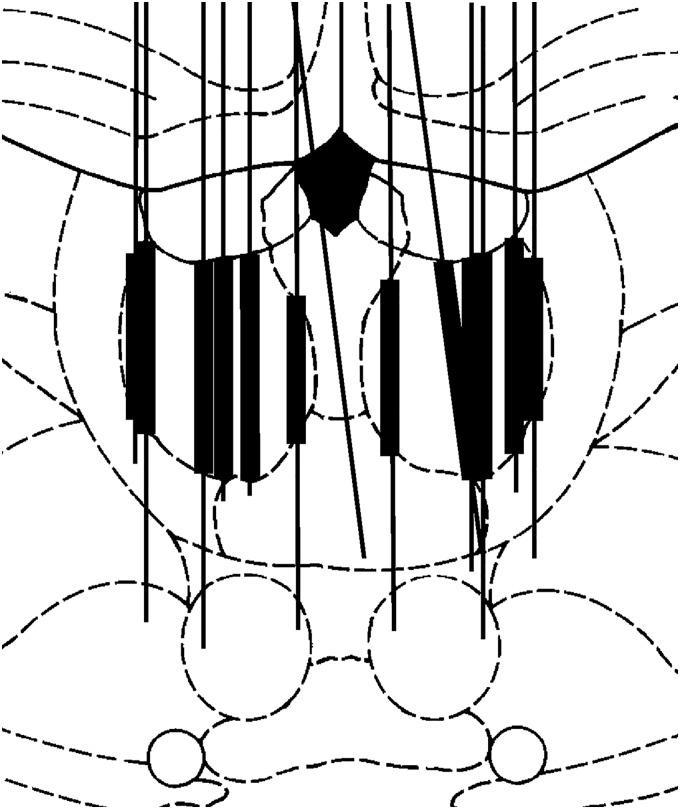
Anatomic locations of tetrode tracks, plotted on a section 6.2 mm anterior to the interaural line in Paxinos and Watson (1998). Narrow lines depict the course of the tracks penetrating through thalamus. Thick lines show the depths at which recorded neurons were assigned to MD and included in this report.

MD activity was recorded after extensive training designed to produce consistent patterns of behavioral responding for dDNMTP and strengthen comparisons with earlier results for mPFC, where rats received equivalent training (Onos et al., 2016). Before surgery, rats were shaped and trained to a criterion of completing 60 trials in a 60-min session with 70% correct on 2 of 3 consecutive days. This required 3-4 mo of daily training (a minimum of 60 training sessions). Tetrode arrays were then surgically implanted. After a week of recovery, water restriction was reinstituted, and rats were trained while neuronal activity was recorded. Data were analyzed only for sessions in which rats completed a minimum of 40 trials in a 60-min session. To sample neurons in different regions of MD, tetrode arrays were implanted at different distances from midline and lowered by one-eighth (0.056 mm) or one-sixteenth of a turn (0.028 mm) for each of the 3 screws after recording sessions when rats performed at criterion for analyzing results or when tetrodes had not been lowered in the previous 3 days. At the end of the study, histologic analyses were conducted to confirm the location of tetrode tracks and determine which neurons were recorded at depths and locations consistent with MD (Fig. 2).

Signals from tetrodes were processed offline for automated cluster cutting to identify signals from individual neurons (Spike Sort 3D, Neuralynx). Cluster plots were rotated manually to identify potential overlap between clusters. Clusters detected by automatic analyses were merged when together they constituted a well-defined cluster in 3D space and exhibited highly similar waveforms at each of the microwires in a tetrode. The criteria for identifying isolated cells were distinct waveforms recorded by different microwires in a tetrode, a well-defined cluster in the 3D plot, a minimum interspike interval >1 ms with an interspike interval histogram peaking >10 ms, signal-to-noise (peak to peak) ratio of 2:1, hyperpolarization that was asymmetrical with depolarization, and an L ratio <1 (Schmitzer-Torbert et al., 2005).

To identify event-related activity consistent with our earlier study of mPFC (Onos et al., 2016), a standard set of 13 averaged peri-event time histograms (PETHs) and raster plots were generated for all isolated neurons using NeuroExplorer. These included PETHs and raster plots aligned relative to TTL pulses marking the four lever press responses, reinforcement events, correct and incorrect choice responses, and delay lever presses when different directions of turning (L versus R) or lever locations (1, 2, 3, or 4) were associated with correct (reinforced) responses. PETHs averaged activity in 200-ms time bins for 5 s before until 5 s after the event marker. Confidence limits for PETH bins were calculated from the frequency of neuronal firing by NeuroExplorer based on the actual Poisson distribution when the expected number of events/bin was <30 and by the Gaussian approximation when more events were predicted.

To distinguish event-related responses without a priori categorization as a particular response type or arbitrarily comparing firing rates across broad epochs of the task (e.g., sample, delay, choice), we defined event-related responses by the minimum criteria of a PETH response with two consecutive 200-ms bins beyond the 99% confidence interval associated with consistent changes in activity in at least 20% of trials shown in raster plots. These criteria are based on several considerations. With 50 bins/10-s epoch, relying on a single bin at *p* < 0.01 as a criterion produces too high a type I error rate. Adopting a more stringent criterion of a single bin at *p* < 0.0002 (the Bonferroni correction for α = 0.01) creates problems with type II errors (missing what seemed to be clear event-related responses). Event-related responses observed for dDNMTP last for multiple consecutive 200-ms time bins in both mPFC (Onos et al., 2016) and MD (present study), consistent with findings for other behavioral tasks (Oyoshi et al., 1996; Jung et al., 1998; Pratt and Mizumori, 2001; Hok et al., 2005; Euston and McNaughton, 2006; Cowen and McNaughton, 2007; Rich and Shapiro, 2009; Totah et al., 2009, 2013; Horst and Laubach, 2012; Watanabe and Funahashi, 2012; Han et al., 2013; Powell and Redish, 2014, 2016; Courtiol and Wilson, 2016). Thus, adopting a criterion of two consecutive bins (rather than one) at *p* < 0.01 provides a means to decrease the probability of a type I error (from random coincidence of activity in successive trials) while minimizing the increase in the probability of a type II error. The requirement of coincident change in at least 20% of trials in a raster plot was intended to prevent robust bursts of activity in a few trials from creating a false positive without creating a constraint that would eliminate spatially restricted firing patterns, which affect firing rates only when behavioral events occur in specific locations (Onos et al., 2016).

Once event-related responses were identified by these criteria, they were categorized using definitions of response types developed in our earlier study of mPFC (Onos et al., 2016). The relative frequencies of different response types were then compared between MD (present results) and mPFC (Onos et al., 2016) using χ^2^ test of independence with standard residuals to identify sources of differences (SPSS statistics 24, IBM). These analyses were restricted to neurons with criterion event-related responses that were classified as a specific response type and for response types for which a minimum of 12 examples were observed between MD and mPFC (to guarantee a minimum expected value of 5 for each cell of the analysis).

To test whether response types are distributed independently across MD neurons, we determined the 95% confidence intervals for the proportions of neurons with each response type in the overall population recorded and used these to estimate the likely number of neurons that should exhibit overlapping responses if they were distributed independently. To do this, we used the modified Wald method recommended by Agresti and Coull (1998) to compute confidence intervals of binomial proportions, *p* = *X*/*n*, as $\tilde{p}\pm z_{\alpha /2}\sqrt{\tilde{p}\left(1-\tilde{p}\right)/\left(n+4\right)},$ where *p̃* = (*X* + 2)/(*n* + 4), with *X* = the number of examples observed and *n* = the number of isolated cells recorded. We then multiplied this interval by the number of neurons classified with other response types to determine the 95% confidence interval for the number of neurons expected to exhibit overlapping response types if all neurons are equally likely to exhibit that response.

We used two approaches to compare firing patterns of populations of neuronal responses classified as the same type in MD (present study) and mPFC (Onos et al., 2016). These analyses were restricted to response types with at least 5 examples in both MD and PFC. Normalized population averages were calculated by converting averaged firing rates to *z*-scores for each 200-ms time bin of individual PETHs based on the overall firing rate of the cell throughout the recording session. These were then averaged separately for MD and mPFC for all cells classified as having the same response type. Normalized firing rates were compared between MD and mPFC for each time bin using two-tailed Mann–Whitney *U* test with Bonferroni correction for multiple comparisons.

PETH results were also used to quantify the timing of event-related activity relative to start, sample, delay, and choice lever press responses. The 99% PETH confidence interval was used to define the beginning and end of each individual response. These analyses were restricted to responses that remained consistently outside the 99th percentile throughout periods of elevated responding (identified in normalized population analyses) to avoid ambiguity defining when responses began and ended. The timing of common responses was compared between MD (present study) and mPFC (Onos et al., 2016) using mixed-model ANOVAs (SPSS statistics 24).

Place field analyses were conducted for neurons with well-isolated action potentials with and without significant event-related activity using NeuroExplorer software to examine the rate of cell firing in a 70-by-70 grid of bins covering the behavioral arena with a minimal time/bin of 0.2 s and minimum of 3 visits in the recording session. Heat maps were generated showing activity averaged throughout a recording session. For selected neurons, additional heat maps were generated using the filter-on-the-fly option to determine the location of rats during periods of elevated activity in PETHs. When rats are trained with two base lever locations in a session, this allows comparison of activity during different directions of movement to or from the base lever.

## Results

Five rats had unilateral arrays of 4 tetrodes, and 4 had bilateral arrays of 2 tetrodes per hemisphere. Histologic analyses revealed tetrode tracks sampling cells from 0.4 to 1.0 lateral from midline, 5.7 to 6.8 mm anterior to IA (Fig 2). Behavioral performance showed little change after recovery from surgery, with all rats performing consistently >60% correct (70% for most sessions) and performing 60 trials within the 60-min session limit for most sessions. Recordings were made from 1634 cells in central thalamus that met criteria for identifying isolated neurons, of which 1179 were at depths consistent with the location of MD (Fig 2). There was a range of 28–229 isolated neurons recorded at depths consistent with MD in individual rats (mean 131.0). These recordings were made during 17–47 recording sessions/rat (mean 31.8). There was an average of 4.1 neurons recorded for each of the 286 sessions when tetrodes were at depths consistent with MD. Criterion event-related activity was observed for 254 (22%) of the 1179 MD neurons, 237 (93%) of which exhibited temporal patterns of event-related activity consistent with response types observed in mPFC for comparably trained rats (Onos et al., 2016). None of the neurons exhibited mixed responses involving more than one response type.

### Similar event-related responses in MD and mPFC

**Figure 3.**
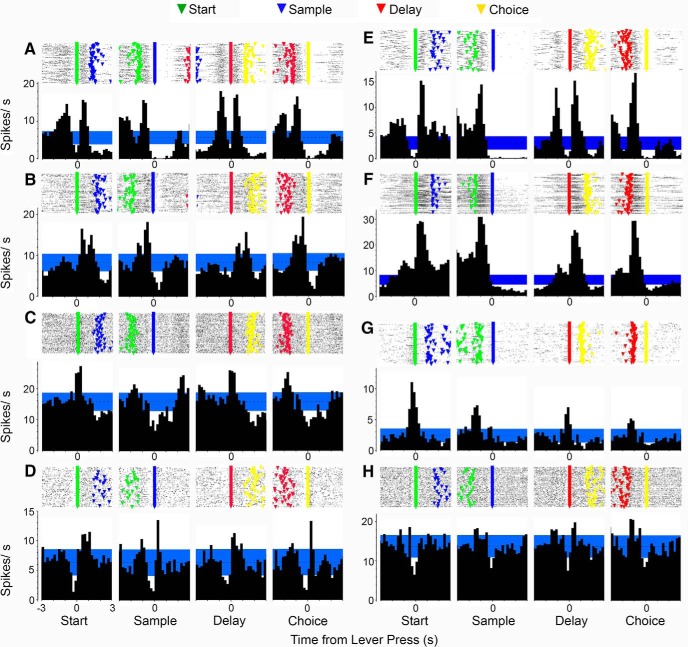
PETHs and raster plots aligned with each of the lever presses in dDNMTP for MD neurons (***A–D***) and previously unpublished examples for mPFC (***E–H***) from Onos et al. (2016) (for additional PFC examples, see Onos et al., 2016). Each plot shows activity from 5 s before to 5 s after the aligned event. Raster plots show event markers for start, sample, delay, and choice lever presses for each trial. Movement 1 responses (***A*** and ***E***) are active before each lever press and inactive following sample and choice responses when rats are stationary while consuming reinforcements delivered to spout immediately above the lever. Movement 2 responses (***B*** and ***F***) are active from start to sample and delay to choice. Base lever responses (***C*** and ***G***) are active for start and delay, but not sample and choice presses. Lever press suppression (***D*** and ***H***) is characterized by reduced activity coincident with each of the lever presses.

Fig. 3 shows PETHs and raster plots aligned with start, sample, delay, and choice lever presses for examples of movement and lever press-related responses observed for both MD (Fig. 3*A–D*) and PFC (Fig. 3*E–H*; unpublished examples from Onos et al., 2016). Movement 1 responses (Fig. 3*A*, *E*) are characterized by increased activity during periods of movement immediately before and after start and delay presses and before, but not after, sample and correct choice responses (rats tend to remain stationary while consuming reinforcers after these presses). Movement 2 responses (Fig. 3*B*, *F*) are characterized by increased activity between start and sample and between delay and choice lever presses. Base lever press responses (Fig. 3*C*, *G*) are associated with increased activity during start and delay, but not sample and choice lever press responses. Lever press suppression (Fig. 3*D*, *H*) is characterized by brief periods of reduced neuronal activity coincident with each of the 4 lever press responses in the dDNMTP sequence.

**Figure 4.**
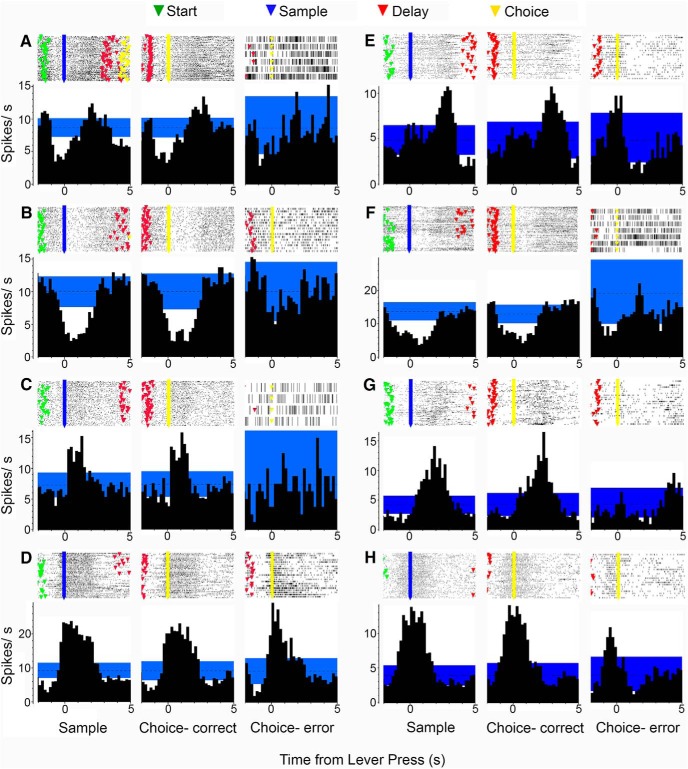
PETHs and raster plots aligned with sample, correct choice, and incorrect choice lever presses in dDNMTP for MD neurons (***A–D***) and previously unpublished examples for mPFC (***E–H***) from Onos et al. (2016) (for additional examples, see Onos et al., 2016). Each plot shows activity from 2 s before until 5 s after the aligned event. Event markers indicate lever presses for individual trials. Reinforcement events consisted of two 0.1-s spurts of water 1 s apart following sample and correct choice presses. Postreinforcement responses (***A*** and ***E***) were characterized by increased activity following reinforcement events. Reinforcement suppression was marked by decreased activity coincident with reinforcement (***B*** and ***F***). Reinforcement excitation responses (***C*** and ***G***) show increased activity concurrent with reinforcement. Reinforcement anticipation responses (***D*** and ***H***) began before lever presses that delivered reinforcement and continued through the reinforcement event for sample and correct choice responses, but not errors when reinforcement was not delivered.

Fig. 4 shows examples of PETHs and raster plots for examples of reinforcement-related responses exhibited by neurons in both MD (Fig. 4*A–D*) and PFC (Fig. 4*E–H*; unpublished examples from Onos et al., 2016) aligned with sample and correct and incorrect choice lever presses. Postreinforcement responses (Fig. 4*A*, *E*) are associated with increased activity after reinforcement, which was delivered in two pulses, one at the lever press and one 1.0 s later. Reinforcement suppression (Fig. 4*B*, *F*) is characterized by decreased activity coincident with sample and correct choice, but not incorrect choice responses. Reinforcement excitation (Fig. 4*C*, *G*) is distinguished by increased activity immediately after reinforced sample and correct choice responses, but not unreinforced incorrect choice responses. Reinforcement anticipation (Fig. 4*D*, *H*) is characterized by increased activity preceding sample and choice responses that persists after reinforcement delivery on sample and correct choice, but not incorrect choice responses.

**Figure 5.**
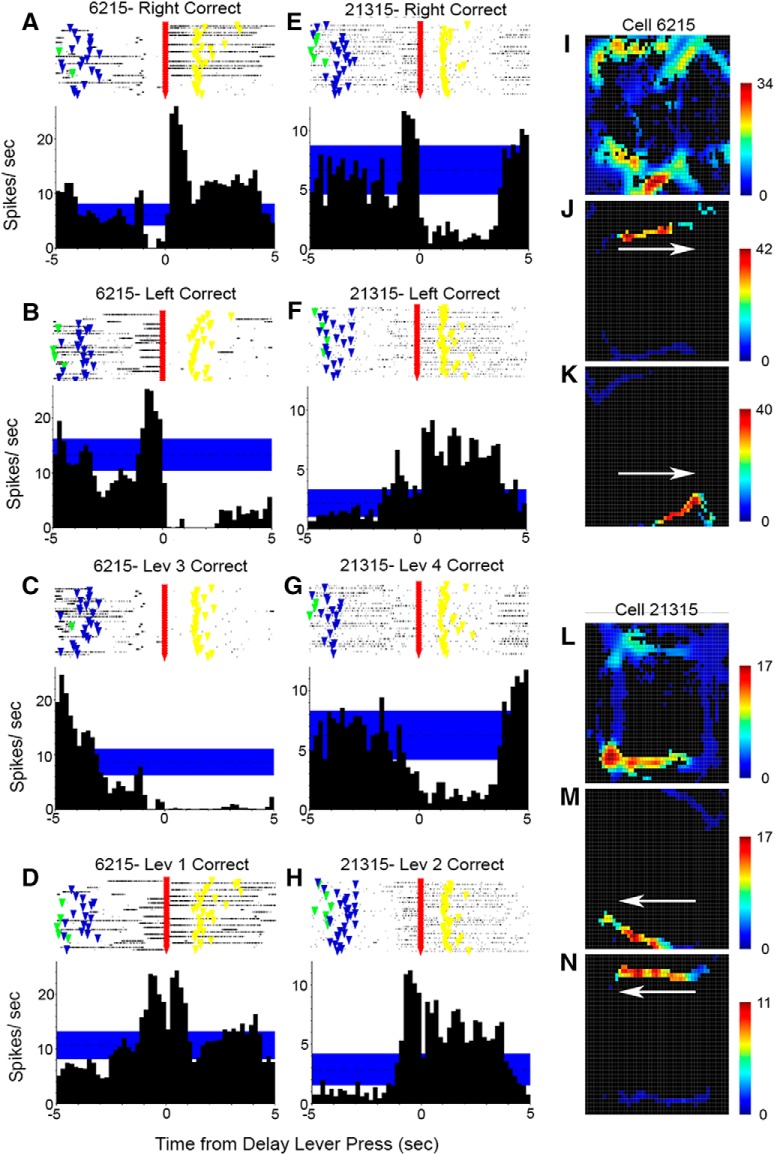
Peri-event raster plots and histograms and heat maps of the spatial distribution of activity for two movement 3 responses recorded in MD for two different animals: ***A–D*** and ***I–K*** for cell 6215 and ***E–H*** and ***L–N*** for cell 21315. The PETH/raster plots are aligned with delay lever presses, showing activity from 5 s before to 5 s after the presses. Results are shown separately for delay presses during trials where correct choices, relative to the base (start/delay) lever, were to the right (***A*** and ***E***), the left (***B*** and ***F***), lever 3 (***C***), lever 1 (***D***), lever 4 (***G***), and lever 2 (***H***). Heat maps show activity averaged for the entire session (***I*** and ***L***), and for 1-s periods of elevated activity immediately before delay for right correct for rat 62 (***J***), after delay for left correct for rat 62 (***K***), before delay for right correct (***M***), and after delay correct for left correct (***N***). Arrows for filter-on-the-fly heat maps (***J***, ***K***, ***M***, and ***N***) indicate the direction of movement associated with elevated activity. Note the offset of trials with elevated activity either before or after the delay press (but not both). The bars to the right of heat maps indicate activity in spikes/s.

We found a movement-related response type not previously observed in PFC (Onos et al., 2016). Movement 3 responses are elevated during movements either toward or away from the delay lever press, specific for both the direction and location of the movement (Fig. 5). These were observed for 11 neurons, 4 at depths consistent with MD, and 7 at depths indicative of the central medial or paracentral nuclei. Movement 3 responses produce distinct PETHs aligned with delay lever presses with elevated activity going into or out of the delay press for trials with left versus right samples (Fig. 5*A* vs. *B*, *E* vs. *F*), with elevated activity during trials with sample responses for a specific lever (Fig. 5*C* vs. *D*, *G* vs. *H*). Spatial maps averaging firing rate throughout the session show elevated activity for specific pathways between levers (Fig. 5*I*, *L*). Filter-on-the-fly maps show that movement 3 responses are elevated during movement in a specific direction over specific paths (Fig. 5*J*, *K*, *M*, *N*; direction indicated by white arrows).

We did not find any neurons that exhibited more than one response type. To determine whether this lack of overlap provides significant evidence for the segregation of different response types in MD, we used the modified Wald method (Agresti and Coull, 1998) to determine 95% confidence intervals for the proportions of different response types in the overall population recorded and used these to estimate the 95% confidence intervals for overlap with other classified response types. Table 1 shows the results of these analyses for the four most frequent response types. For each of these responses, the lower bound of the 95% confidence interval indicates that there should have been 1 or more cases of overlap (range 1.6–9.3). These results indicate that it is likely that there is some degree of segregation of neurons exhibiting different response types in MD. Because no cases of overlap were observed, these results do not rule out the possibility that there is complete segregation of different response types in MD.

**Table 1.** Expected overlap of response types for responses in the MD.

Response	Modified Wald 95% confidence interval for population proportion	Expected overlap with other response types
Movement 1	0.079 ± 0.015	9.3 to 13.7
Movement 2	0.014 ± 0.007	1.6 to 4.7
Reinforcement anticipation	0.025 ± 0.010	3.1 to 7.3
Reinforcement excitation	0.041 ± 0.011	5.7 to 9.9

Modified Wald population confidence intervals are based on all isolated neurons recorded in MD. The expected overlap indicates the number of neurons exhibiting other responses types that should also exhibit the response type in question if response types are distributed independently across MD neurons.

**Figure 6.**
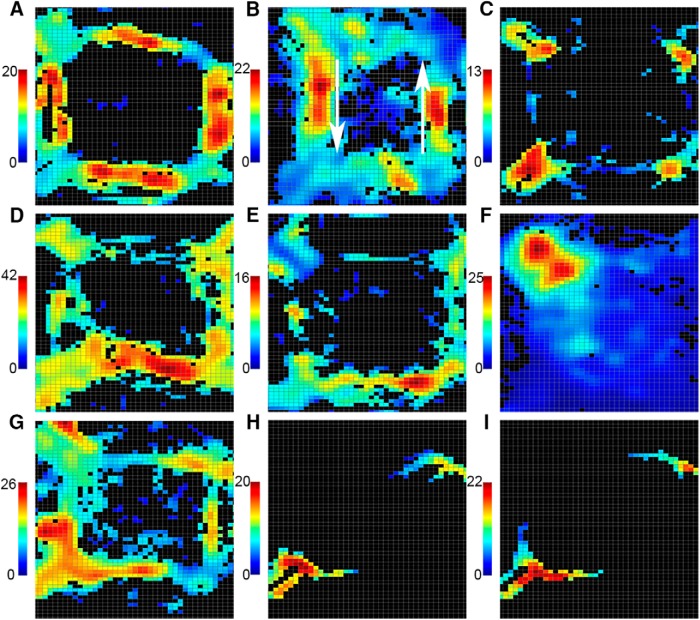
Examples of spatial heat maps. Movement 1 responses (***A***) were associated with increased activity along pathways traversed between levers during dDNMTP. Some movement 2 responses resembled results for mPFC (Onos et al., 2016) with increased activity in one direction (in this case left) along pathways from base levers to sample or choice levers (***B***; arrows show directions of movement associated with increased firing). Reinforcement excitation was associated with increased activity at locations of drinking spouts (immediately above levers) where water reinforcers were delivered (***C***). The example here was from a session in which the base lever was selected from the 4 alternatives, and thus reinforcement was delivered at 4 locations corresponding with areas of increased activity. Some neurons (33/244, 14%) showed more restricted patterns of activation, for instance movement 1 (***D***) or movement 2 (***E***) responses, with elevated firing along a specific pathway, or reinforcement excitation (***F***), with elevated activity in one specific location of reinforcement. Many movement 2 responses had areas of activation along pathways traversed as well as at sample and choice levers (***G***). Filter-on-the-fly analyses showed that rats had reached locations of these levers at the time of maximal activity 1 s before sample (***H***) and choice (***I***) responses. The bars to the left of heat maps indicate activity in spikes/s.

Video tracking was available for 244 of 254 cells with and 680 of 925 without criterion PETH responses. Heat maps showing the spatial distribution of neuronal activity throughout recording sessions revealed patterns of activity consistent with event-related analyses. Thus movement-related responses were generally associated with elevated activity along pathways between levers, whereas reinforcement-related responses were associated with elevated activity at locations where reinforcement was delivered (Fig. 6*A–C*). Movement 2 responses provided an exception to this, where event-related activity was increased near locations of reinforcement where these movements ended (Fig. 6*G*). Filter-on-the-fly heat maps restricted to periods of elevated activity in PETHs showed that this activity occurred at locations of reinforcement after movements to sample or correct choice levers (Fig. 6*H*, *I*). These results seem consistent with evidence from timing analyses (see below) that movement 2 responses occurred later in MD than in mPFC. Like mPFC (Onos et al., 2016), there was variability in spatial-tuning of MD responses. Some (33/244, 14%) with classified response types had areas of elevated activity restricted to 50% or less of areas where a behavioral event occurred (Fig. 6*D–F*). Also like mPFC (Onos et al., 2016), spatially restricted fields were observed for 75/680 (11%) neurons that did not exhibit a classified response type.

**Figure 7.**
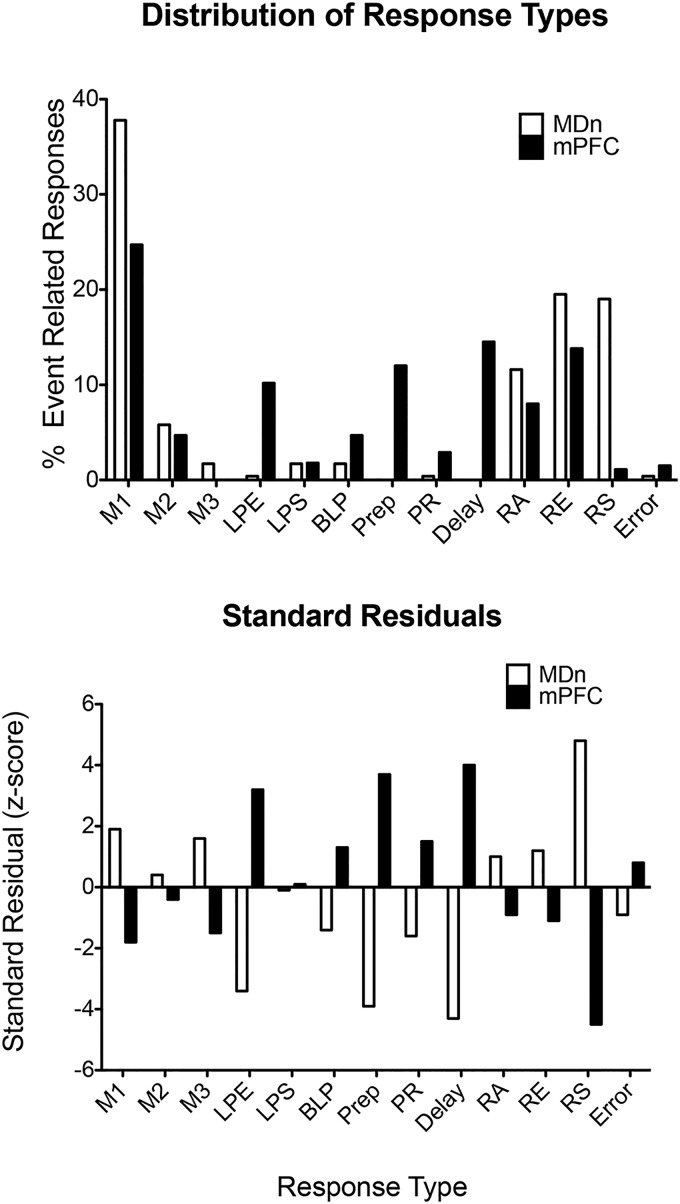
The upper figure shows the percentage of neurons that met criteria for movement 1 (M1), movement 2 (M2), lever press excitation (LPE), base lever press (BLP), delay-related (Delay), reinforcement anticipation (RA), reinforcement excitation (RE), and reinforcement suppression (RS), in MD (present study) and mPFC (data from Onos et al., 2016). These response types account for 231/254 (90.9%) criterion event-related responses in MD and 258/293 (88.1%) in mPFC. The lower figure shows standard residuals from χ^2^ analyses of these data plotted as *z*-scores. Positive residuals represent response types that were more frequent in one area than the other.

Comparison with mPFC neurons revealed substantial differences in relative numbers of different response types in thalamus and cortex (Fig. 7). We found no preparatory responses (increased activity during the intertrial interval ending within 1.0 s of the start lever press), no delay-related responses (beginning during the sample response reinforcement event and persisting until after the delay lever press), and only 1 example of lever press excitation (increased activity coincident with each of the four lever press responses in each dDNMTP trial). In contrast, there was a greater preponderance of movement and reinforcement-related responses in MD than observed earlier in mPFC (Onos et al., 2016). A χ^2^ test of independence compared relative frequencies of 9 response types that met the minimum requirement of 12 examples in combined data for MD and mPFC (Fig. 7). These response types accounted for 231/254 (90.9%) criterion event-related responses in MD and 258/293 (88.1%) in mPFC. The results showed that the distribution of these response types differed between MD and mPFC, Pearson χ^2^ = 144.6 (df = 8), *p* < 0.0001, with all cells having expected values >8. Standard residual analyses identified lever press excitation, preparation, delay, and reinforcement suppression as the main sources of the significant χ^2^ results (Fig. 7, standard residual *z* score >3 in one area and <3 in the other).

### Population analyses of common event-related response types

**Figure 8.**
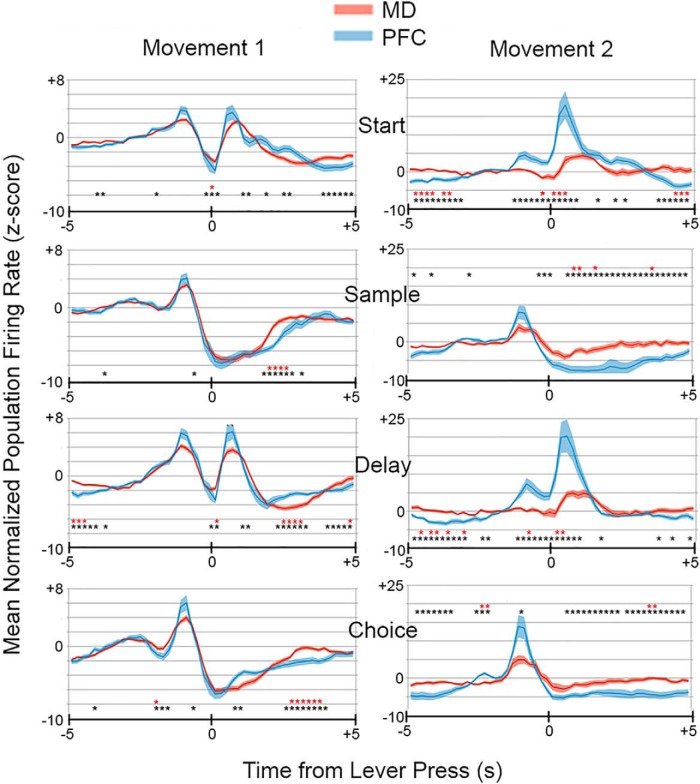
Normalized population responses for movement 1 and 2 responses in MD and PFC. Results are plotted from 5 s before until 5 s after start, sample, delay, and choice lever presses in 200-ms time bins. Results show average activity of 91 neurons in MD and 68 in mPFC with movement 1 responses and 14 neurons in MD and 13 in mPFC with movement 2 responses. Black asterisks indicate time bins that differed at *p* < 0.05 and red asterisks bins that differed at *p* < 0.001 (Bonferroni correction for α = 0.05) for two-tailed Mann–Whitney tests. Thin dark lines represent the mean; wider light colored lines the SEM.

**Figure 9.**
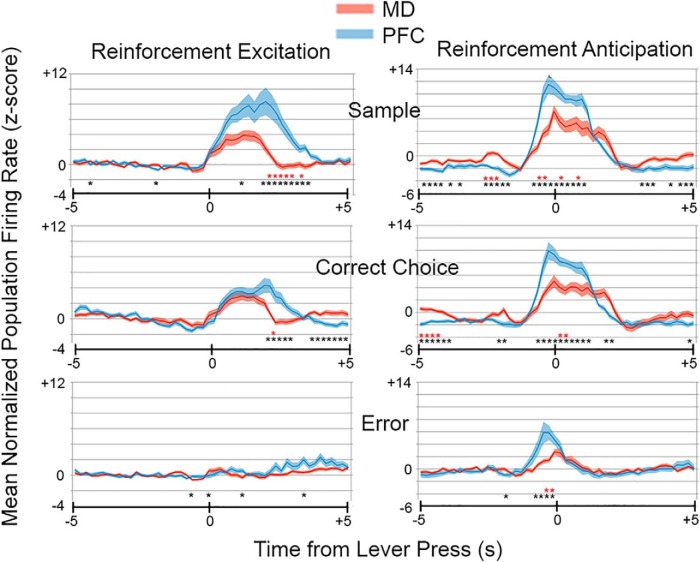
Normalized population responses for reinforcement excitation and anticipation responses in MD and PFC. Results are plotted from 5 s before until 5 s after sample, correct choice, and incorrect choice lever presses in 200-ms time bins. Results show average activity of 47 neurons in MD and 38 in mPFC for reinforcement excitation and 28 neurons in MD and 22 in mPFC for reinforcement anticipation. Black asterisks indicate time bins that differed at *p* < 0.05, and red asterisks, bins that differed at *p* < 0.001 (Bonferroni correction for α = 0.05) for two-tailed Mann–Whitney tests. Thin dark lines represent the mean; wider light colored lines the SEM.

We observed sufficient numbers of responses in MD (present study) and mPFC (Onos et al., 2016) to conduct population analyses of four response types: movement 1 (MD: *n* = 91; PFC: *n* = 68), movement 2 (MD: *n* = 14, PFC: *n* = 13), reinforcement excitation (MD: *n* = 47; PFC: *n* = 38), and reinforcement anticipation (MD: *n* = 28; PFC: *n* = 22). Figs. 8 and 9 show normalized average population activity for these responses along with asterisks marking time bins where the two-tailed Mann–Whitney test revealed a difference between MD and mPFC at *p* < 0.05 (black, for descriptive purposes) and *p* < 0.001 (red, to mark significant differences with the Bonferroni correction for α = 0.05).

Movement 1 population responses in both MD and mPFC showed sharp ramping up then down of activity before and after start and delay responses and before sample and choice responses (Fig. 8). Examination of individual responses revealed that the bursts of action potentials after start and delay responses were the same as those observed before sample and choice responses, as rats moved to the next press in the dDNMTP sequence following a brief pause (Fig. 3). Activity dropped below *z* = 0 after each of these bursts, with a more robust and prolonged decrease after sample and choice responses when rats consume reinforcements delivered immediately above the response lever. Movement 2 population responses in MD and mPFC were associated with bursts of activity as rats moved from start to sample and from delay to choice. Here there were striking differences in temporal patterns of activity in mPFC and MD. Movement 2 responses began in mPFC 1.4 s before start and delay lever presses, ramping up coincident with these presses, and ramping down 1.2 s later (Fig. 8). These differed from movement 1 responses in remaining elevated (*z* > 0) during start and delay lever presses. Movement 2 responses began in MD 0.2 s after start and delay lever presses and ended 1.2 s later (1.4 s after these presses). Mann–Whitney analyses of movement 2 population responses showed significantly higher activity (*p* < 0.001) in mPFC during periods of elevated activity (*z* > 0 for both groups) and significantly lower activity during periods of diminished activity (*z* < 0 for both groups). These trends are indicative of enhanced signal-to-noise in mPFC for movement 2 responses. Results for movement 1 responses were more mixed. There were no significant group differences during periods of elevated activity, but some during periods of diminished activity where MD > mPFC and where mPFC > MD (Fig. 8).

Population analyses of reinforcement excitation revealed increases in activity in MD and mPFC coincident with sample and correct choice lever presses when reinforcement was delivered, but not for incorrect choice presses when it was not (Fig. 9). The main difference between these areas was the longer persistence of reinforcement excitation activity in mPFC. This was confirmed by Mann–Whitney analyses that showed significant differences between MD and mPFC during extended periods of reinforcement excitation in mPFC. Reinforcement anticipation began and ended earlier than reinforcement excitation for both MD and mPFC (Fig. 9), increasing above *z* = 1.2–0.8 s before sample and choice lever presses for MD and PFC and falling below *z*=0 at 2.0 s after sample and correct choice and 0.8 s after incorrect choices for MD, compared to 1.6 s for sample and correct choices and 0.6 s for errors for mPFC. Mann–Whitney analyses revealed significant group differences (*p* < 0.001) during common periods of elevated activity when mPFC > MD and during common periods of diminished activity with MD > mPFC. This pattern is consistent with results for movement 2 responses (above) and is indicative of enhanced signal-to-noise.

**Figure 10.**
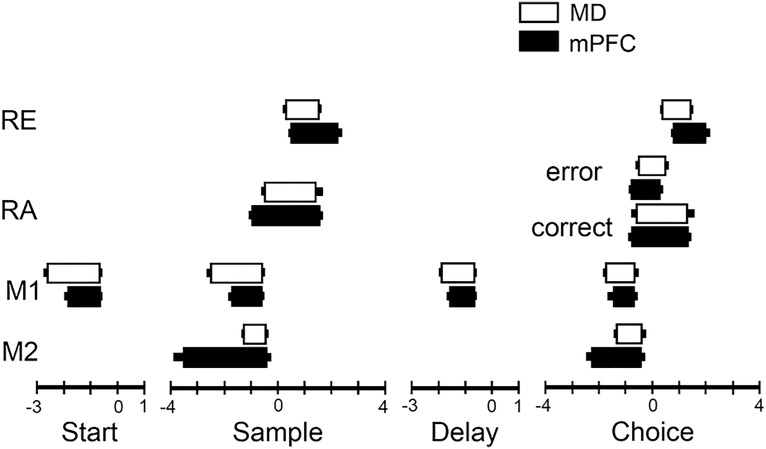
Temporal duration of reinforcement excitation (RE), reinforcement anticipation (RA), movement 1 (M1), and movement 2 (M2) in MD thalamus and mPFC. Thick bars represent the mean duration and thin bars the SEM. Separate results are plotted for RA for correct choices (followed by reinforcement) and errors (not followed by reinforcement). Onset was measured from when responses increased above the 99% confidence interval and offset from when responses fell below this level in individual PETH plots. These analyses were restricted to neurons that remained outside the 99% confidence interval throughout common periods of elevated activity identified in normalized population plots (see Figs. 8 and 9).

Response time (RT) analyses were based on the beginning and end of individual neuronal responses defined by the 99% confidence interval for PETH. To avoid ambiguity when PETHs fluctuated back and forth across this limit during common periods of elevated activity, these analyses were restricted to neurons with responses that were outside this limit throughout the response (defined by normalized population functions, see Figs. 8 and 9). RTs were analyzed for 68/91 MD and 49/68 PFC neurons that exhibited movement 1 responses (Fig. 10). These responses began earlier in MD (2.22 s before presses vs. 1.66 s for mPFC) and for earlier presses in the dDNMTP sequence (2.30 s before start presses vs. 2.15 s for sample, 1.85 for delay, and 1.63 for choice). A mixed-model two-factor ANOVA revealed significant differences in time of onset for recording site (MD vs. PFC), *F*_1,115_ = 12.853, *p* < 0.001; press (preceding start, sample, delay, and choice lever presses), *F*_3,345_ = 14.368, *p* < 0.001; and for the interaction of these factors, *F*_3,345_ = 3.742, *p* = 0.011. In contrast, the offset of movement 1 responses showed little difference between recording sites and across lever presses. Thus movement 1 responses became shorter throughout the dDNMTP sequence and showed closer correspondence between MD and mPFC at offset than onset (Fig. 10). A mixed-model two-factor ANOVA showed no significant effect on movement 1 offset for recording site, *F*_1,115_ = 2.302, *p* = 0.132, press, *F*_3,345_ = 1.939, *p* = 0.123, or the interaction of these factors, *F*_3,345_ = 1.016, *p* = 0.386.

RTs were analyzed for 12/14 neurons in MD and 13/13 in PFC that exhibited movement 2 responses. These began earlier in mPFC (2.91 s before sample/choice presses vs. 1.35 s for MD) and earlier before the sample than the choice presses (2.33 vs. 1.81 s) in mPFC (Fig. 10). There was little difference in the offset of movement 2 response in MD or mPFC or preceding sample or choice. Two-factor mixed-model ANOVAs of movement 2 onset revealed significant differences for site (MD vs. mPFC), *F*_1,23_ = 23.505, *p* < 0.001, press (preceding sample versus choice), *F*_1,23_ = 14.075, *p* = 0.001, and the interaction of these factors, *F*_1,23_ = 23.505, *p* < 0.001). None of these effects were significant for movement 2 offset (all *F* < 1).

RTs were analyzed for 34/47 neurons in MD and 29/38 in mPFC exhibiting reinforcement excitation responses. These responses tended to start earlier in MD (0.32 vs. 0.56 s for mPFC after reinforced lever presses) and earlier after sample than correct choice presses (0.36 vs. 0.53 s). Two-factor mixed-model ANOVA showed significant effects of press (sample vs. correct choice), *F*_1,61_ = 11.597, *p* = 0.001 the interaction of time and recording site, *F*_1,61_ = 7.015, *p* = 0.010, but not for recording site (MD vs. mPFC), *F*_1,61_ = 3.074, *p* = 0.085. Analyses of reinforcement excitation offset showed significant effects of recording site, *F*_1,61_ = 11.813, *p* = 0.001, and time, *F*_1,61_ = 7.008, *p* = 0.010, but not for their interaction, *F* < 1.

RTs were analyzed for 26/28 neurons in MD 17/17 in mPFC with reinforcement anticipation responses. Reinforcement anticipation tended to begin earlier in mPFC (0.82 s before reinforced lever presses vs. 0.55 s for MD) and preceding sample presses (0.75 vs. 0.56 s for correct choice presses). Two-factor mixed-model ANOVA revealed a significant effect of press (sample vs. correct choice), *F*_1,41_ = 4.508, *p* = 0.040, but not recording site (MD s. mPFC), *F*_1,41_ = 2.960, *p* = 0.093, or the interaction of these factors, *F* < 1. There were minimal differences in the offset of reinforcement anticipation between MD and mPFC and for sample and correct choice responses. ANOVA did not reveal significant effects of site (*F*_1,41_ = 1.357, *p* = 0.251), press (*F* < 1), or their interaction (*F*_1,41_ = 1.230, *p* = 0.274).

## Discussion

To elucidate interactions between MD and mPFC in decision-making, we recorded MD neurons in rats performing a dDNMTP task, using carefully matched training procedures to compare results with a previous study of mPFC (Onos et al., 2016). We found criterion event-related responses for 22% (254/1179) of neurons recorded in MD, 93% of which (237/254) exhibited response types observed in mPFC. Compared with mPFC, MD had a preponderance of neurons with responses related to movement (45% vs. 29%) and reinforcement (51% vs. 27%), few with responses coincident with all lever presses (0.4% vs. 10.2%) or base lever presses at the beginning of sample and delay phases of dDNMTP (1.7% vs. 4.7%), and none with preparatory or delay-related activity. No neurons were observed with more than one response type. Analyses of proportions of response types in the population of neurons recorded showed that this lack of overlap was outside the 95% confidence interval for the four most frequent response types. These findings indicate that the distributions of different response types are not independent and thus provide evidence that there is some degree of segregation of these response types in MD. Analyses of averaged population firing patterns and individual neuronal RTs revealed similar temporal patterns of activity for common response types in MD and mPFC, some of which differed in onset and offset relative to specific behavioral events. These results suggest that MD and mPFC interact processing information related to actions and outcomes in mPFC-dependent decision-making.

### Representation of actions and outcomes in MD

In mPFC, dDNMTP responding is represented by distinct populations of neurons that fire in conjunction with preparation, lever presses, movements, delay, and reinforcement (Onos et al., 2016). In MD, movement-related responses included neurons firing during all periods of movement (movement 1), specifically during movements to sample and choice levers (movement 2), and during movements in defined directions and locations during specific movement sequences (movement 3). Onos et al. (2016) observed movement 1 and 2, but not movement 3, responses in cingulate, prelimbic, and infralimbic areas of mPFC. Movement 3 responses were observed at depths consistent with paralaminar MD and paracentral and central medial nuclei, areas that receive dense projections from medial agranular cortex (Groenewegen, 1988; Vertes, 2002), a cortical area implicated in integrative sensorimotor function (Reep and Corwin, 1999; Bailey and Mair, 2004, 2007). The limited number of MD neurons firing in conjunction with lever presses and the lack of preparatory- or delay-related activity suggests that MD–PFC interactions are less important for functions mediated by these responses.

Population and RT analyses showed similar temporal patterns of activity for common response types, with the exceptions that movement 2 responses began earlier and reinforcement excitation ended later in mPFC in both analyses, and movement 1 responses began later in mPFC in RT, but not population analyses (Figs. 8, 9, and 10). It is not clear whether the differences between analyses for movement 1 onset reflect the restriction of RT analyses to robust responses with unambiguous onsets and offsets. Movement 2 population responses increased above baseline rates (*z* > 2.0) in mPFC 1.4 s before start and 1.2 s before delay responses and remained elevated above this level through the subsequent lever press when activity increased sharply. In MD, these responses did not increase above baseline until 0.4 s after start and 0.2 s after delay responses. The early increase in mPFC population activity was confirmed by Mann–Whitney analyses (Fig. 8). RT analyses showed earlier onsets of movement 2 responses (activity above the 99th percentile for individual neurons) in mPFC for both start (0.08 vs. 0.72 s for MD) and delay (–0.02 vs. 0.40 s) responses. Population analyses of reinforcement excitation revealed activity elevated above baseline in mPFC from 0.0 to 3.4 s after sample (vs. 0.2–2.0 s for MD) and from 0.4 to 2.6 s after correct choices (vs. 0.4–2.0 s for MD). The prolonged activation of reinforcement excitation in mPFC neurons was confirmed by Mann–Whitney analyses (Fig. 9). RT analyses showed no significant difference in onset, but a later offset for reinforcement excitation in mPFC, lasting 0.40–2.25 s after sample response (vs. 0.32–1.64 s for MD) and 0.73–1.96 s after correct choices (vs. 0.36–1.46 s for MD).

The present study did not include task manipulations that would allow us to determine whether reinforcement-related activity reflects information about reinforcement per se or reinforcement-related motor responses. The possibility of a simple motor-related correlation is difficult to reconcile with the prolonged firing for reinforcement excitation in mPFC compared with MD, as well as the time course of reinforcement anticipation responses, which began as rats moved toward reinforcement-related levers and lasted until after reinforcement events ended (Figs. 9 and 10). Our finding of reinforcement-related activity for 51% of classified MD neurons seems consistent with evidence from behavioral studies that MD lesion or inactivation eliminates biasing effects of rewards or reward-related cues on action selection and the ability to adapt to changes in action-outcome contingencies (Ostlund and Balleine, 2008; Leung and Balleine, 2015; Parnaudeau et al., 2015).

Previous studies have shown that neuronal responses in rat MD represent information about spatial location in both delayed alternation (Han et al., 2013) and two-alternative odor discrimination (Courtiol and Wilson, 2016). Here we examined spatial coding properties by videotracking movements throughout training sessions and generating heatmaps comparing activity in areas traversed during training sessions. Our results were comparable to similar analyses of mPFC (Onos et al., 2016). All neurons with event-related responses showed activity patterns consistent with those responses: for instance, movement 1 responses are most active on pathways between levers or excitatory reinforcement responses at locations where reinforcement is delivered (Fig. 6*A–C*). Some neurons exhibited more restricted spatial maps, for instance, movement 1 responses firing selectively on specific segments of pathways traversed between levers or reinforcement excitation at a single location (Fig. 6*D–F*). Movement 2 responses occurred later in MD than mPFC (Fig. 8), typically after rats reached levers for sample or choice responses (Fig. 6*G–I*). As a result, evidence of directionally specific firing for most of these neurons was lacking [see Fig. 8 in Onos et al. (2016) for examples of directional tuning in mPFC]. The discovery of movement 3 responses in MD (Fig. 5) provides evidence of highly specific contextual coding, representing information about both the direction (R versus L) and location of movement, specific for when that movement occurs within a dDNMTP trial.

### Influence of MD on PFC function

Interactions between MD and PFC are fundamentally important for adaptive decision-making (Parnaudeau et al., 2013; Browning et al., 2015; Mitchell, 2015; Bolkan et al., 2017), although it is uncertain how MD influences PFC activity during goal-directed behavior. PFC has diverse connections with sensory, motor, and limbic systems that provide rich sources of information about sensory features of the external world, motor function, and internal goals and motivations (Miller and Cohen, 2001; Hoover and Vertes, 2007; Barbas, 2015; Barbas and García-Cabezas, 2016). PFC is also innervated by several higher-order (cortical-recipient) thalamic nuclei where lesions impair PFC function (Bailey and Mair, 2005; Mair et al., 2015; Mitchell, 2015; Vertes et al., 2015; Aggleton et al., 2016). MD is the main source of thalamic driver input to middle layers of PFC (Giguere and Goldman-Rakic, 1988; Groenewegen, 1988). MD is driven by projections from layer 5 of PFC, amygdala, piriform cortex, and superior colliculus and receives substantial inhibitory inputs from reward and motor-processing areas of pallidum and the thalamic reticular nucleus (Kuroda and Price 1991a,b; Ray et al., 1992; Zikopoulos and Barbas 2007; Timbie and Barbas 2015; Root et al., 2015; Vertes et al., 2015). Although neurons in first-order (subcortically driven) thalamic nuclei have been shown to mirror the activity of their driver inputs—for instance, lateral geniculate nucleus and retinal ganglion cells—there is much less known about higher-order thalamic neurons that receive at least some of their driver inputs from cortical layer 5 pyramidal cells (Sherman, 2016). Here, we found many neurons in MD with movement- and reinforcement-related responses that closely resemble mPFC response types, but few with responses related to lever press actions and none with responses related to preparation or delay that are also observed in mPFC during dDNMTP (Onos et al., 2016). These results suggest that MD has specific effects on mPFC function related to movement and reward during dDNMTP. They provide evidence of a higher-order thalamic nucleus with neural activity that mirrors activity patterns observed for neurons in reciprocally connected areas of cerebral cortex.

Early studies emphasized delay-related activity in MD of monkeys performing working memory tasks such as DNMTP that resembled the time courses of delay-related activity in dorsolateral PFC (Fuster and Alexander, 1971, 1973; Tanibuchi and Goldman-Rakic, 2003; Watanabe and Funahashi, 2004a,b, 2012). Interestingly, these MD responses were eliminated when dorsolateral PFC was inactivated by cooling (Alexander and Fuster, 1973). More recently Bolkan et al. (2017) used optogenetic methods in mice to show that T-maze DNMTP is impaired by inhibition of connections between MD and mPFC during trials with 60-s, but not 10-s, memory delays. Inhibition of MD to mPFC projections produced significant impairment when applied during the delay phase and inhibition of mPFC to MD when applied during the choice phase, suggesting distinct functional effects for afferent and efferent components of these reciprocal connections. Bolkan et al. (2017) reported confirmatory evidence that MD activity leads mPFC during the delay phase, whereas mPFC leads MD during the choice phase. Analyses of delay-related activity of single neurons in mPFC of these mice did not reveal individual cells with responses comparable to delay-related responses in primates that are sustained throughout memory delays and represent information about preceding samples or subsequent choices (Watanabe and Funahashi, 2012). The DNMTP impairments observed by Bolkan et al. (2017) are inconsistent with the literature showing significant impairment for PFC or MD lesions in rodents, nonhuman primates, and human subjects for working memory tasks with much shorter (≤10-s) delays (Isseroff et al., 1982; Zola-Morgan and Squire, 1985; Freedman and Oscar-Berman, 1986; Goldman-Rakic, 1987; Young et al., 1996; Bailey and Mair, 2005; Watanabe and Funahashi, 2012). It is not clear whether these results reflect unique properties of T-maze DNMTP, where movements are restricted to specific pathways and odors from preceding occupancy and consumption of food rewards in the sample arm provide transient, nonmnemonic cues indicating correct choices. It is also unclear whether deficits associated with such long memory delays reflect a failure of working memory maintenance or the ability to sustain attention or motivation across lengthy trials.

Onos et al. (2016) observed delay-related responses in mPFC during dDNMTP at short delays (≤5 s) consistent with delay-related activity in primates (Fuster and Alexander, 1971, 1973; Tanibuchi and Goldman-Rakic, 2003; Watanabe and Funahashi, 2004a,b, 2012) and delays associated with behavioral impairment after MD or mPFC lesions in rats (Young et al., 1996; Bailey and Mair, 2004, 2005). Here, we found no evidence of delay-related activity in MD that parallels delay responses observed for dDNMTP in mPFC. The lack of delay-period responses in MD is consistent with findings for spatial delayed alternation in rats (Han et al., 2013), although it is inconsistent with evidence from primates, in which task-related neural responses persist in individual MD and PFC neurons throughout memory delays (Watanabe and Funahashi, 2012). It is unclear whether the finding of delay-related activity in monkey MD reflects species differences or the tasks used to assess working memory in these studies. We found numerous neurons in MD that exhibited event-related activity, closely correlated with mPFC response types, that were not delay-related. Others have described diverse event-related responses in MD outside memory delays in rats performing other behavioral tasks (Oyoshi et al., 1996; Han et al., 2013; Courtiol and Wilson, 2016). Whether MD is critical for maintaining information in working memory or not, it seems clear that MD supports a wider range of function during goal-directed behavior.

Sherman and Guillery have argued that the main driving inputs from cortex to higher-order thalamic nuclei are branches of axons innervating downstream motor systems that provide a potential source of efference copy (or corollary discharge) signaling information about impending, self-generated actions (Guillery and Sherman, 2011; Sherman, 2016). Sommer and Wurtz (2006) report that projections from superior colliculus to MD provide presaccadic efference signals to MD that affect visuo-spatial processing in frontal eye field neurons in monkeys. Watanabe and Funahashi have shown that MD has a larger proportion of neurons than dorsolateral PFC that represent prospective motor information about forthcoming saccades in monkeys during delay and response periods of ocular delayed response tasks (Watanabe and Funahashi, 2004a,b, 2012; Watanabe et al., 2009). Here, we found that a larger proportion of neurons in MD than mPFC correlated with movements and reinforcement during dDNMTP. Because choice in dDNMTP is defined by directional movements between levers, these results seem consistent with the predominance of neurons representing information about forthcoming saccades in ocular delayed-response tasks where choice is defined by directional eye movements.

Although other interpretations exist, response types observed here exhibit properties consistent with a purely motor efference copy function (Guillery and Sherman, 2011). Movement 2 responses begin before associated movements and represent the direction of subsequent movement from start to sample and from delay to choice (Onos et al., 2016). Movement 1 responses start later and do not exhibit directional specificity. Although they provide little information about forthcoming movements, they consistently predict subsequent lever press responses. Reinforcement anticipation responses similarly predict forthcoming consummatory behavior. These begin as rats approach levers where a press can potentially produce reinforcement and persist when reinforcement is delivered but not when it is not (Figs. 9 and 10). Reinforcement excitation begins later, coincident with delivery of reinforcement in population responses (Fig. 9) and 0.32–0.73 s later in RT analyses (Fig. 10). These provide a reliable indication of when rats pause to consume reinforcements before either the delay phase of that trial or the start of the next trial. Although these results are consistent with the view that MD monitors motor output, it is unclear how such a function could account for the effects of MD lesions on associative learning, decision-making, or updating information about reward value (Bradfield et al., 2013; Mitchell, 2015; Chakraborty et al., 2016).

In the rat, descending projections from layer 5 pyramidal cells in mPFC innervate MD as well as subcortical limbic, autonomic systems, and motor areas (Vertes, 2004; Gabbot et al., 2005; Xiao et al., 2009). It thus seems reasonable to extend Sherman and Guillery’s logic and hypothesize that MD is specialized to monitor signals from PFC related to autonomic and limbic function, as well as motor function. Consistent with this view, MD receives robust projections from limbic cortices, ventral pallidum, amygdala, and the ventral tegmental area that provide privileged access to systems mediating reinforcement and reinforcement-guided responding (Mitchell, 2015; Root et al., 2015; Vertes et al., 2015; Khani and Rainer, 2016). Lesion and inactivation studies have shown that MD is important for learning instrumental contingencies between actions and outcomes and for adaptive decision-making requiring frequent updating of outcome values or action-outcome contingencies (Ostlund and Balleine, 2008; Bradfield et al., 2013; Parnaudeau et al., 2013, 2015; Mitchell, 2015; Chakraborty et al., 2016). Our results show precisely timed responses related to movement and reinforcement in MD after extensive dDNMTP training. The close temporal correspondence with neuronal responses in mPFC (Figs. 8, 9, and 10) is indicative of the strong reciprocal connections between these areas. The preponderance of MD responses related to movement and reinforcement suggest that these connections are primarily concerned with actions and outcomes underlying choice within the context of the dDNMTP task. Our results are consistent with evidence from previous recording studies that MD neurons represent task-relevant information about discriminative stimuli, previous (retrospective) and forthcoming (prospective) actions, and action-outcomes consistent with its importance for adaptive decision-making (Oyoshi et al., 1996; Watanabe and Funahashi, 2012; Yu et al., 2012; Han et al., 2013; Courtiol and Wilson, 2016).

## Synthesis

Reviewing Editor: Tim Bussey, University of Cambridge Decisions are customarily a result of the Reviewing Editor and the peer reviewers coming together and discussing their recommendations until a consensus is reached. When revisions are invited, a fact-based synthesis statement explaining their decision and outlining what is needed to prepare a revision will be listed below. The following reviewer(s) agreed to reveal their identity: Matthew Roesch, Donald Katz.

Reviewer 1

This study examines single neuron recordings from mediodorsal thalamus (MD) in rats performing a working memory task. The authors have previously recorded from medial prefrontal cortex (mPFC) in the same task. The two areas are compared to each other. The goal was to determine if firing was similar to PFC. It is an important question because MD and mPFC have reciprocal connections, which are thought to be critical for various executive functions, including working memory. Further it is important work because few have recorded from MD and mPFC in the same exact task. The authors find many neurons that fire around the same time as neurons in mPFC do, however they claim to find a novel movement type response in MD that represented both direction and location. MD had fewer response patterns related to lever press, preparation, and memory compared to mPFC.

I had many concerns. I think the authors did a good job revising the manuscript to address my concerns. I particularly like the new population histograms. Like reviewer 2, I thought the original manuscript described a laundry list of possible responses and that the counts of neurons showing task-related effects were somewhat low and overstated. The paper has definitely improved in both these aspects. However, I am surprised that the authors cannot split data up into direction and correctness. Surely interesting difference between MD and PFC related to function and performance on the rats would come from such an analysis.

Reviewer 2

I was very pleased with the authors' response to my previous critique, and to the changes in the manuscript. In particular, I chuckled at their response regarding the Chi-squared test-I am delighted and unsurprised that they did the appropriate test...particularly given that 4 out of 5 researchers (in my experience) do not! Anyway, the paper is more modest and clean now, and in my opinion is well worthy of publication.

My only continued request has to do with the assortment of response types, and in particular the point that is made that neurons typically respond to only 1 feature of the task. The analysis that I was asking for is one that asks whether, taking into account the probability of finding each type of response in and of itself, it is actually surprising that so little overlap was found. My suspicion is that they will find that there isn't actually a significant trend toward actual segregation of response types, but rather that their results reflect a random overlap of response types.
